# FOXO3A‐short is a novel regulator of non‐oxidative glucose metabolism associated with human longevity

**DOI:** 10.1111/acel.13763

**Published:** 2023-01-08

**Authors:** Evan E. Santo, Rasmus Ribel‐Madsen, Peter J. Stroeken, Vincent C. J. de Boer, Ninna S. Hansen, Maaike Commandeur, Allan A. Vaag, Rogier Versteeg, Jihye Paik, Ellen M. Westerhout

**Affiliations:** ^1^ Department of Pathology & Laboratory Medicine Weill Cornell Medicine New York New York USA; ^2^ The Novo Nordisk Foundation Center for Basic Metabolic Research Clinical Pharmacology Copenhagen Denmark; ^3^ The Danish Diabetes Academy Odense Denmark; ^4^ Department of Oncogenomics, Academic Medical Center University of Amsterdam Amsterdam The Netherlands; ^5^ Human and Animal Physiology Wageningen University Wageningen The Netherlands; ^6^ Department of Biomedical Sciences, Endocrinology and Metabolism University of Copenhagen Copenhagen Denmark; ^7^ Department of Clinical Sciences, Clinical Research Centre Lund University Malmö Sweden

**Keywords:** aging, FOXO, FOXO3A, glycolysis, insulin, PI3K, skeletal muscle, SNP

## Abstract

Intronic single‐nucleotide polymorphisms (SNPs) in FOXO3A are associated with human longevity. Currently, it is unclear how these SNPs alter FOXO3A functionality and human physiology, thereby influencing lifespan. Here, we identify a primate‐specific FOXO3A transcriptional isoform, FOXO3A‐Short (FOXO3A‐S), encoding a major longevity‐associated SNP, rs9400239 (C or T), within its 5′ untranslated region. The FOXO3A‐S mRNA is highly expressed in the skeletal muscle and has very limited expression in other tissues. We find that the rs9400239 variant influences the stability and functionality of the primarily nuclear protein(s) encoded by the FOXO3A‐S mRNA. Assessment of the relationship between the FOXO3A‐S polymorphism and peripheral glucose clearance during insulin infusion (Rd clamp) in a cohort of Danish twins revealed that longevity T‐allele carriers have markedly faster peripheral glucose clearance rates than normal lifespan C‐allele carriers. In vitro experiments in human myotube cultures utilizing overexpression of each allele showed that the C‐allele represses glycolysis independently of PI3K signaling, while overexpression of the T‐allele represses glycolysis only in a PI3K‐inactive background. Supporting this finding inducible knockdown of the FOXO3A‐S C‐allele in cultured myotubes increases the glycolytic rate. We conclude that the rs9400239 polymorphism acts as a molecular switch which changes the identity of the FOXO3A‐S‐derived protein(s), which in turn alters the relationship between FOXO3A‐S and insulin/PI3K signaling and glycolytic flux in the skeletal muscle. This critical difference endows carriers of the FOXO3A‐S T‐allele with consistently higher insulin‐stimulated peripheral glucose clearance rates, which may contribute to their longer and healthier lifespans.

## Graphical abstract

Genetic studies of humans have identified the gene FOXO3A to be associated with longevity. Here, we describe how a primate‐specific form of FOXO3A which is regulated by a FOXO3A locus longevity variant functions to control insulin sensitivity in the skeletal muscle. This discovery demonstrates a potentially causative relationship between increased glucose clearance and longer life in humans and provides a molecular tool for further discovery of genes regulating human longevity.

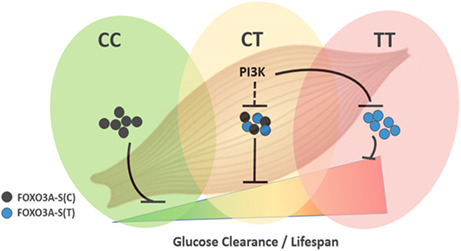

## 1 INTRODUCTION

The insulin/PI3K/Akt/FOXO signaling pathway is a powerful regulator of longevity in model organisms and has also been implicated in the determination of human lifespan. Suppression of insulin signaling *in Caenorhabditis elegans* and Drosophila causes FOXO activation and robust FOXO‐dependent lifespan extension (van Heemst, 2010). In mice, suppression of IGF/insulin signaling has resulted in lifespan extension as well, confirming the evolutionarily‐conserved importance of this pathway in longevity (Bluher et al., 2003; Selman et al., 2008). More directly, it has recently been shown that deletion of FOXO1/3/4 in the mouse nervous system results in accelerated age‐dependent axonal degeneration (Hwang et al., 2018). This finding highlights the importance of FOXO in the suppression of aging in the mammalian nervous system. In humans, multiple genetic association studies have linked SNPs at the FOXO3A locus to human longevity (Bae et al., 2018; Broer et al., 2015; Flachsbart et al., 2009, 2017; Joshi et al., 2017; Li et al., 2009; Soerensen et al., 2010; Sun et al., 2015; Willcox et al., 2008). Significantly, all of the longevity‐relevant FOXO3A variants are outside of the known protein‐coding sequences of FOXO3A (Flachsbart et al., 2013). Additionally, SNPs in FOXO1, FOXO4, and FOXO6 are not associated with longevity (Kleindorp et al., 2011). These previous findings suggest that the FOXO3A locus and/or gene products may perform a non‐redundant function in human longevity.

As a downstream mediator of insulin signaling, FOXOs are also of central importance in the maintenance of whole‐body glucose homeostasis. FOXO1 acts cooperatively with FOXO3 and FOXO4 in the liver to activate gluconeogenesis when insulin signaling is reduced, thereby maintaining blood glucose levels (Haeusler et al., 2014). Recently, the FOXO3A pro‐longevity genotype has been associated with increased insulin sensitivity (Banasik et al., 2011; Sun et al., 2015). In further support of this association between insulin sensitivity and longevity in humans, The Leiden Longevity Study found that peripheral but not hepatic insulin sensitivity is positively associated with familial longevity (Wijsman et al., 2011). Additionally, increased insulin sensitivity among centenarians has been observed when compared to their younger controls (Paolisso et al., 2001). In a recent GWAS study for body mass index (BMI) and associated traits, the pro‐longevity rs9400239 genotype was associated with lower BMI (*p* = 1.61e‐08), smaller waist circumference (*p* = 1.66e‐07), smaller hip circumference (*p* = 1.14e‐03), less coronary artery disease (*p* = 2.32e‐03), and lower systolic blood pressure (*p* = 4.97e‐03) (Locke et al., 2015). This lower BMI may help explain why the FOXO3A pro‐longevity genotype has also been associated with improved self‐rated health at old age, reduced coronary heart disease, and reduced all‐cause mortality (Willcox et al., 2016, 2017; Zettergren et al., 2018). The FOXO3A pro‐longevity genotype has also been associated with increased activity of daily living (ADL) and decreased bone fracture within a Danish oldest‐old cohort; this is particularly true for rs9400239 (ADL *p* = 0.02) (Soerensen et al., 2015). Taken together, these previous findings demonstrate that FOXO3A longevity variants and rs9400239, in particular, are not only associated with increased lifespan but improved healthspan and that enhanced insulin sensitivity might be a central phenotype underlying the health benefits associated with the pro‐longevity FOXO3A genotype.

So far, no mechanisms have been discovered that link any of the FOXO3A longevity variants to tissue‐specific FOXO3A functions of relevance to human longevity. Here, we describe a primate‐specific transcript arising from the human FOXO3A locus, FOXO3A‐Short (FOXO3A‐S), which contains the rs9400239 longevity SNP within its 5’ UTR and is primarily expressed in the skeletal muscle. Although both alleles of the FOXO3A‐S mRNA encode constitutively nuclear n‐terminally truncated forms of the FOXO3A protein, the protein products have allele‐dependent differences in stability and functionality. Specifically, the pro‐longevity FOXO3A‐S T‐allele (3A‐S(T)) produces a protein product of reduced stability which is unable to suppress glycolytic flux in the skeletal muscle in the presence of active PI3K signaling, while the non‐longevity FOXO3A‐S C‐allele (3A‐S(C)) is more stable and able to suppress glycolytic flux in both the presence and absence of active PI3K signaling. We find these differences between the alleles to be of great consequence in a cohort of human subjects where insulin‐stimulated peripheral glucose clearance is markedly higher in subjects heterozygous and homozygous for the pro‐longevity 3A‐S(T) allele. Collectively, these findings identify non‐oxidative glucose metabolism within the skeletal muscle as a particularly important metabolic pathway influencing human longevity.

## 2 RESULTS

### 2.1 FOXO3A‐Short encodes a human longevity SNP and is highly expressed in the skeletal muscle

**FIGURE 1:**
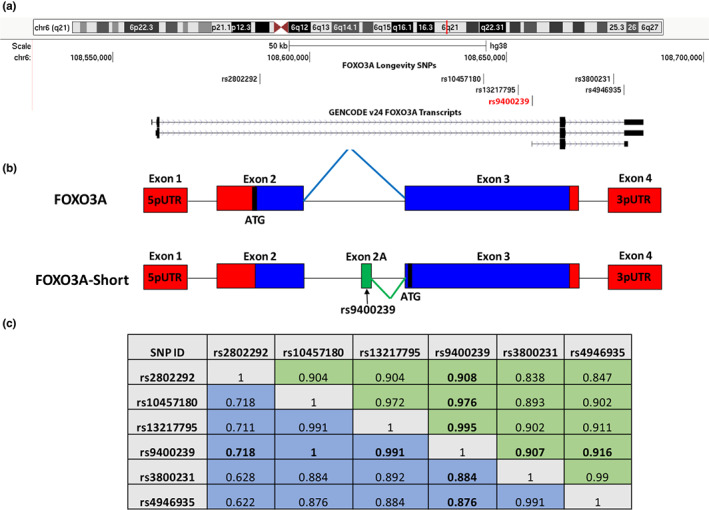
The FOXO3A locus encodes a novel transcript which contains a longevity SNP. (a) A genomic view of the FOXO3A locus rendered by the UCSC Genome Browser with FOXO3A transcripts and lead longevity SNPs annotated (Hg38). (b) A non‐scaled cartoon representation of the known FOXO3A coding exons and splice junction in blue, UTRs in red and the novel exon and splice junction that comprise FOXO3A‐short in green. Start codons (ATG) for each isoform are indicated. (c) Linkage disequilibrium (LD) analysis of the FOXO3A longevity SNPs using data supplied from the 1000 Genomes Project (Phase 3 Version 5) and represented as correlation coefficients (*r* ^2^) generated using the LDmatrix tool (https://ldlink.nci.nih.gov/?tab=ldmatrix). Linkages for Europeans are highlighted in blue, East Asians are highlighted in green.

To gain insight into potential alternative functions for the FOXO3A longevity SNPs, we utilized the UCSC Genome Browser to visualize the mapping of the longevity SNPs and transcripts to the FOXO3A locus (Figure 1a). A short transcript consisting of an uncharacterized first exon (subsequently called exon 2A) which is transcribed from within intron 2 and spliced into exon 3 of canonical FOXO3A was evaluated. Interestingly, the longevity SNP rs9400239 was found to map within exon 2A (Figure 1a; Figure 1b). The minor T‐allele of rs9400239 has been strongly associated with longevity in two Caucasian cohorts (Flachsbart et al., 2009; Soerensen et al., 2010) but has not been examined in Asian longevity studies. To gain a better understanding of the association of rs9400239 with human longevity, we assessed its linkage disequilibrium (LD) with other lead FOXO3A longevity SNPs by using haplotype data from the 1000 Genomes Project. A longevity GWAS meta‐analysis identified rs10457180 as the lead FOXO3A SNP in Caucasians (Broer et al., 2015). Additional studies independently identified rs4946935 as their lead FOXO3A SNP in Caucasian cohorts (Bae et al., 2018; Flachsbart et al., 2017). A large parent‐based longevity GWAS discovered rs3800231 in a cohort of primarily Caucasian background (Joshi et al., 2017). In Asian populations, rs2802292 has been well‐studied but is of less significance in Caucasian populations (Li et al., 2009; Sun et al., 2015; Willcox et al., 2008). The lead SNP in Asians seems to be rs13217795 (Sun et al., 2015). We find rs9400239 to be in remarkably tight LD with all of these lead SNPs in Asians (*r* ^2^ > 0.9 for all comparisons); in Caucasians, this is true for all (*r* ^2^ > 0.87) except rs2802292 (*r* ^2^ = 0.718, Figure 1c). From this analysis, we conclude that the rs9400239 SNP encoded within exon 2A is strongly associated with human longevity across populations.

**FIGURE 2:**
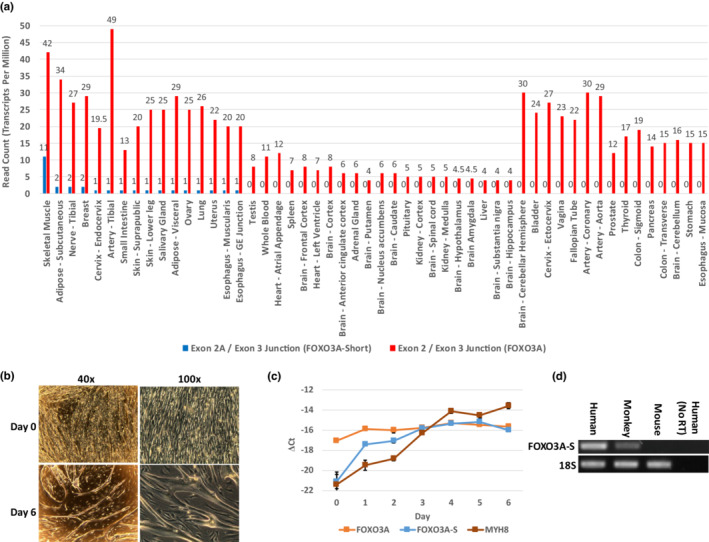
FOXO3A‐S is predominantly expressed in the skeletal muscle. (a) GTEx version 8 (https://www.gtexportal.org) human tissue mRNA expression data for both FOXO3A and FOXO3A‐S depicted in TPM (transcripts per million). Data labels above each bar are the exact TPM value. (b) Representative light microscopy images from a differentiation time series of the human myoblast line LHCNM2. Day 0 is under normal myoblast growth conditions and Day 6 is under differentiation conditions. (c) RT‐qPCR measurement of FOXO3A, FOXO3A‐S, and MYH8 expression from the LHCNM2 differentiation time course imaged in (b) represented as ∆Ct. Error bars are SD. (d) RT‐PCR of pooled skeletal muscle mRNA from multiple individuals in multiple species for FOXO3A‐S transcript using primers designed to conserved genomic sequence. 18S rRNA served as a loading control.

In order to determine the mRNA expression pattern of this novel transcript throughout human tissues, we utilized data from the GTEx project. Using the “transcript browser” tool, we were able to query the expression of both canonical FOXO3A (exon 2/exon 3 splice junction) and the novel transcript (exon 2A/exon 3 splice junction) individually (GTEx version 8; https://gtexportal.org). This analysis revealed that the novel transcript is predominantly expressed in the skeletal muscle while being nearly absent in all other tissues (Figure 2a). In contrast, FOXO3A is ubiquitously expressed (Figure 2a). We further tested expression of the transcript in the immortalized human myoblast cell line LHCNM2, which can be differentiated into myotubes in vitro (Figure 2b) (Zhu et al., 2007). RT‐qPCR of a differentiation time course of these cells for FOXO3A, the differentiation marker MYH8, and the exon 2A transcript showed robust FOXO3A expression in both myoblasts and myotubes. In contrast, MYH8 and the exon 2A transcript were nearly absent in myoblasts but became robustly expressed in myotubes (Figure 2c). Temporally, the exon 2A transcript was expressed early in myoblast differentiation and remained high in mature myotubes, corroborating the observed expression in primary human skeletal muscle. 5’ RACE using the LHCNM2 myotube mRNA as a template confirmed that the novel mRNA has a 5′ m^7^g cap and that exon 2A is 242 bp long with the entire spliced product being 1643 bp (exclusive of the 3’ UTR). Having noticed that the sequence conservation between exon 2A and the mouse genome was very minimal (data not shown), we designed RT‐PCR primers to the available conserved sequence between mouse and human within exon 2A and exon 3. Performing RT‐PCR in pooled skeletal muscle cDNA from human, monkey, and mouse revealed that the exon 2A‐initiated transcript most likely does not exist in mice but does exist in monkeys (Figure 2d). From these findings, we conclude that the exon 2A‐initiated transcript represents a genuine primate‐specific transcript which we refer to as FOXO3A‐Short (FOXO3A‐S).

### 2.2 The two rs9400239 variants of the FOXO3A‐S transcript encode proteins of differential stability

**FIGURE 3:**
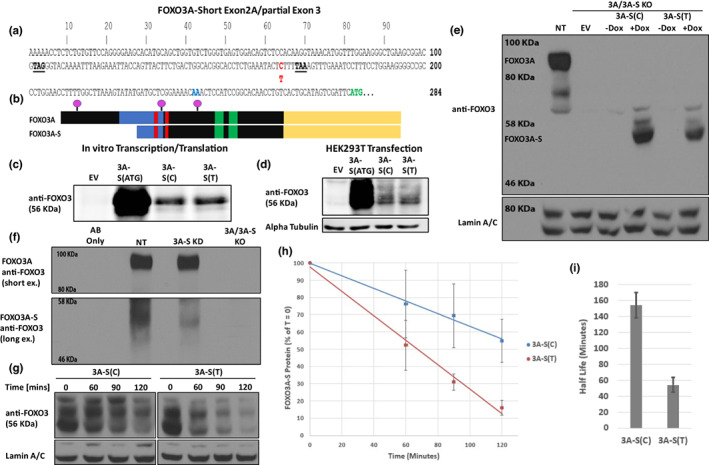
The FOXO3A‐S transcript encodes different FOXO3A‐related proteins depending on the genotype of the rs9400239 SNP. (a) The complete sequence of exon 2A of FOXO3A‐S and partial sequence of the common FOXO3A/FOXO3A‐S exon 3. Highlighted in red is the rs9400239 SNP (C/T), in green the putative ATG start codon for FOXO3A‐S and in blue the splice junction between exon 2A and exon 3. In bold/underline are stop codons in‐frame with the putative ATG start/canonical FOXO3A reading frame. (b) Annotation of the FOXO3A‐S protein sequence relative to FOXO3A. The forkhead domain is colored blue, nuclear localization signals are red, nuclear export signals are green, the transactivation domain is yellow, and the three Akt phosphorylation sites are purple. (c) Western blot of the products of in vitro transcription/translation reactions performed with FOXO3A‐S clones. Two of the clones are full‐length FOXO3A‐S transcripts containing either rs9400239 variant (3A‐S(C) and 3A‐S(T)) ‐ the rs9400239 variant being the only difference between them. The third clone is one starting exactly from the first ATG (3A‐S(ATG)) which truncates the rs9400239 position and remaining 3’ UTR. (d) Western blot from HEK293T transfections of the same clones used in (c). (e) Western blot of LHCNM2 myotube lysates (Day 7) derived from either control (NT) or a FOXO3A/FOXO3A‐S‐targeting spJCRISPR construct single‐cell clone that was further transduced with Dox‐inducible empty vector (EV), 3A‐S(C) or 3A‐S(T) clones and then treated or not with 0.1 μg/ml Dox on Day 5. Nuclear Lamin A/C is shown as a loading control. (f) Western blot of immunoprecipitations of endogenous FOXO3A and FOXO3A‐S from LHCNM2 myotube lysates (Day 7) collected from spJCRISPR single‐cell clones. All myotube cultures were treated with 200 nM of the proteasome inhibitor Bortezomib for 18 h (starting Day 6) prior to harvest. (g) Western blots of lysates from cycloheximide (CHX) pulse‐chase experiments performed with the LHCNM2 lines from (e). Myoblasts were treated with 0.1 μg/ml Dox for 48 h post‐seeding, pulsed with 300 μM CHX then harvested at indicated time points (T = 0 no CHX). Western blots are representative of three independent experiments. (h) Plot of the band densitometry quantification of Western blots from (g). FOXO3A‐S protein levels were first normalized to corresponding Lamin A/C controls which were then normalized to T = 0 within each series. Plots are average of three replicate experiments with error bars being SD. (i) FOXO3A‐S protein half life calculated by averaging the computed half lives from each time point within (h). Error bars are SD.

We next investigated the protein coding potential of the FOXO3A‐S transcript. Inspection of the full‐length FOXO3A‐S cDNA identified the first ATG 282 bp downstream of the transcriptional start site (TSS). This ATG is Met221 of canonical FOXO3A; therefore, in‐frame with the canonical FOXO3A stop codon (Figure 3a). The rs9400239 SNP is located 164 bp downstream of the TSS and 4 bp upstream of a stop codon that is in‐frame with the ATG (Figure 3a). This stop codon ensures that the rs9400239 SNP is part of the FOXO3A‐S 5’ UTR because it prevents rs9400239 from participating in any FOXO3A‐related protein coding sequence. Translation from this ATG yields a predicted protein of 453 aa with a predicted mW of 48 KDa. The predicted FOXO3A‐S protein encodes a partially truncated forkhead domain but still retains the nuclear localization and export signals as well as the transactivation domain (Figure 3b) (Greer & Brunet, 2005). Despite the partial forkhead domain loss, the crystal structure of DNA‐bound FOXO3A suggests that FOXO3A‐S may bind to DNA via a retained C‐terminal coil (amino acids 236–255 in FOXO3A) (Tsai et al., 2007).

To assess the protein‐coding potential experimentally, full‐length FOXO3A‐S transcripts containing both rs9400239 variants (3A‐S(C) and 3A‐S(T)) were cloned as well as a clone starting from the first ATG (3A‐S(ATG)). These clones were first tested in in vitro transcription/translation reactions. Western blot analysis with an antibody raised against the far c‐terminus of FOXO3A yielded primarily single‐protein bands for all clones running around 56 KDa (Figure 3c). Notably, the bands derived from the full‐length clones were much less intense than that achieved with the 3A‐S(ATG) clone. These same clones were also transfected into HEK293T cells, which gave the same band intensity pattern on Western blot as the cell‐free system (Figure 3d). However, multiple bands were observed for each clone in the HEK293T lysates, suggesting post‐translational modification of the FOXO3A‐S protein (Figure 3d). The large amount of protein produced using the 3A‐S(ATG) clone as opposed to either full‐length FOXO3A‐S clone strongly suggests that the 5′ end of the mRNA may be greatly inhibitory of translation.

We next sought to identify the endogenous FOXO3A‐S protein. To facilitate these experiments, we first deleted all endogenous FOXO3A and FOXO3A‐S using a spJCRISPR construct targeting the splice acceptor site of exon 3 that is common to both FOXO3A and FOXO3A‐S. We also generated a construct to specifically delete FOXO3A‐S while sparing FOXO3A; this construct targeted the splice donor site of FOXO3A‐S exon 2A. LHCNM2 myoblasts were infected with both constructs, and single‐cell clones for each construct were isolated, differentiated, and screened by Western blot for FOXO3A knockout and RT‐qPCR for FOXO3A‐S knockout. We succeeded in generating a clone that was completely null for both FOXO3A and FOXO3A‐S (3A/3A‐S KO; Figure 3e; Figure S1). The FOXO3A‐S‐specific construct only yielded a clone with partial FOXO3A‐S knockdown (3A‐S KD; Figure S1). To ensure that the FOXO3A‐S protein could be unambiguously identified on Western blot in the LHCNM2 myotube background, we established two sub‐lines by infecting the 3A/3A‐S KO clone with either the 3A‐S(C) or 3A‐S(T) transcripts under the control of a doxycycline‐inducible promoter. With Dox addition, three bands were apparent in both lines with the primary band running at 56 KDa (Figure 3e). We then performed immunoprecipitations (IPs) from myotube lysates derived from lines “no target” (NT) control, 3A‐S KD and 3A/3A‐S KO using crosslinked FOXO3A c‐terminal antibody and detecting with rabbit conformation‐specific secondary antibody to avoid heavy chain detection. These IPs resulted in the identification of an endogenous band running at 56 KDa that was greatly reduced in the 3A‐S KD line and completely absent in the 3A/3A‐S KO line; corroborating the FOXO3A‐S RT‐qPCR in these lines (Figure 3f; Figure S1). It is notable that this band is not detectable by direct Western blotting of myotube lysates (Figure 3e & data not shown) and can only be visualized by IP/Western blot. This corroborates our findings that FOXO3A‐S is a poorly‐translated protein from the full‐length transcripts (Figure 3c,d) and therefore of relatively low abundance. From these experiments, we concluded that we successfully identified the endogenous FOXO3A‐S protein; demonstrating that the FOXO3A‐S transcript is a bona fide protein‐coding gene.

We next assessed the stability of the FOXO3A‐S protein to see if there were mechanisms additional to poor translation regulating FOXO3A‐S protein abundance. For this, we performed cycloheximide (CHX) chase assays in LHCNM2 myoblasts overexpressing either the 3A‐S(C) or 3A‐S(T) transcripts in the 3A/3A‐S KO background (Figure 3g,h). Incredibly, we found the half life of the 3A‐S(C)‐derived proteoform to be on average 154 min, while the half life of the 3A‐S(T)‐derived proteoform was 54 min (Figure 3i; *p* = 0.0007). This remarkable 2.8‐fold stability difference strongly indicates that the proteoforms derived from each allele might be distinct proteins. Another possibility is that there are multiple proteoforms encoded by both transcripts, perhaps produced at different ratios to each other depending on the allelic variant. Although the 3A‐S(C)‐derived proteoform is relatively more stable than that from 3A‐S(T), both are highly unstable proteins; as the median half life of all proteins in mouse C2C12 myotubes is 43 h (Cambridge et al., 2011). From these analyses, we conclude that the rs9400239 longevity SNP directly alters the identity and/or composition of the FOXO3A‐S translation products and that all forms are highly unstable and inefficiently translated.

### 2.3 The FOXO3A‐S‐encoded SNP rs9400239 is associated with peripheral glucose clearance in vivo

**FIGURE 4:**
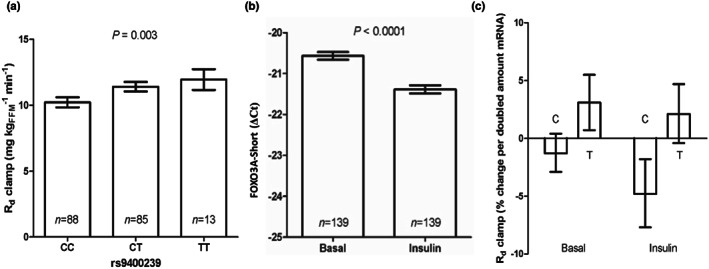
The FOXO3A‐S T‐allele is positively associated with peripheral glucose clearance in vivo. (a) Association of the rs9400239 genotype with the Rd clamp glucose disposal parameter. The association was calculated using a mixed‐ANOVA regression analysis correcting for age, sex, BMI, twin pair, and zygosity. (b) RT‐qPCR for FOXO3A‐S mRNA from muscle biopsy pairs harvested before (Basal) and during insulin infusion (insulin) in 139 healthy individuals. Expression was calculated relative to 18S rRNA using the ∆Ct method. Error bars are expressed as SEM, and the significance was calculated using the two‐tailed paired Student's t‐test. (c) Allele‐specific RT‐qPCR of FOXO3A‐S mRNA under basal and insulin‐stimulated conditions correlated with Rd clamp. The expression of each allele was corrected to 18S rRNA using the ∆Ct method. A mixed ANOVA regression was used to assess the correlation between allele‐specific values and Rd clamp correcting for age, sex, and BMI. Error bars are 95% CI.

A recent study showed that familial longevity was positively associated with the rate of peripheral glucose clearance (Wijsman et al., 2011). In humans, peripheral glucose clearance is primarily a function of the skeletal muscle (Shulman et al., 1990). Given the near‐exclusive expression of FOXO3A‐S in the skeletal muscle and the known roles of FOXOs in insulin signaling, we hypothesized that FOXO3A‐S may regulate peripheral glucose clearance and that this function may be influenced by the rs9400239 longevity SNP. Previously, a study of a metabolically well‐characterized cohort of Danish twins found a moderate association (*β* = 0.85; *p* = 0.04) between one of the FOXO3A longevity SNPs, rs2802292, and peripheral glucose clearance as measured by Rd clamp (Banasik et al., 2011). SNP rs9400239 was not included in this study, so we decided to genotype the same study cohort of 186 individuals for rs9400239 and repeated the analysis. The rs9400239 SNP showed a much stronger association (*β* = 1.2; *p* = 0.003) with Rd clamp, the longevity T‐allele being associated with faster clearance (Figure 4a). Notably, these associations with glucose clearance parallel the associations of these SNPs with longevity in Caucasians: rs9400239 is more strongly associated with both peripheral glucose clearance and longevity in Caucasians than rs2802292 (Figure 1c; average *r* ^2^ = 0.938 for all lead FOXO3A longevity SNPs except rs2802292 when compared with rs9400239 in Caucasians; average *r* ^2^ = 0.67 for rs2802292 when compared to all lead SNPs except rs9400239 in Caucasians). This suggests a genetic co‐mapping of these traits within the FOXO3A locus and raises the possibility that the increased peripheral glucose clearance of rs9400239 T‐allele carriers may be at least partly responsible for the increase in longevity.

Within a subset of this cohort (139 individuals), paired muscle biopsies had been harvested from all individuals before (basal) and during insulin infusion, which allowed us to explore the possibility of FOXO3A‐S mRNA expression being associated with the genotype and/or peripheral glucose clearance. We first checked for a correlation between FOXO3A and FOXO3A‐S mRNA levels and found a strong correlation under both basal and insulin‐infused conditions (Figure S2a; basal *r* ^2^ = 0.8088 *p* < 0.00001, insulin *r* ^2^ = 0.7515 *p* < 0.00001). Previously, analysis of mRNA isolated from these samples using a FOXO3A‐specific Taqman probe showed that FOXO3A mRNA levels were down‐regulated during insulin infusion (*p* < 0.0001) (Banasik et al., 2011). A similar down‐regulation was observed for FOXO3A‐S mRNA levels (*p* < 0.0001; Figure 4b). Despite this acute regulation between conditions, we did not find a significant association between FOXO3A or FOXO3A‐S mRNA levels and Rd clamp (data not shown). Furthermore, we did not find a significant correlation between the genotype of SNP rs9400239 and the levels of FOXO3A or FOXO3A‐S mRNA, neither before or during insulin infusion (data not shown). To corroborate this lack of correlation between the rs9400239 genotype and canonical FOXO3A expression, we queried FOXO3A mRNA expression vs. the rs9400239 SNP in the skeletal muscle using the publicly available GTEx resource and found no relationship (GTEx version 8; https://gtexportal.org, *p* = 1). This finding highlights that the rs9400239 SNP functions differently than those FOXO3A longevity SNPs previously reported to be regulating FOXO3A mRNA expression (Flachsbart et al., 2017; Grossi et al., 2018).

Although we did not find a correlation between total FOXO3A‐S mRNA levels and Rd clamp, we decided to quantitate FOXO3A‐S allele expression specifically across the cohort by developing an allele‐specific RT‐qPCR method (Figure S2b–d). We hypothesized this might reveal quantitative relationships between the FOXO3A‐S mRNA variants and phenotypic traits as we suspected the proteins encoded by each allele to be biochemically distinct. Again, we found no significant correlation between the genotype of SNP rs9400239 and the mRNA levels of the C or T‐alleles of FOXO3A‐S (Figure S2e), corroborating our findings from the total FOXO3A‐S RT‐qPCR. We next tested the relationship between the mRNA levels of the individual FOXO3A‐S C and T‐alleles and glucose clearance rates. Multivariate analysis correcting for age, sex, and BMI showed that the mRNA levels of the FOXO3A‐S C‐allele during insulin challenge were inversely correlated with the rate of glucose clearance (*β* = −0.46, *p* = 0.005); under basal conditions, there was no correlation (Figure 4c). In contrast, the T‐allele transcript levels did not show any correlation to the rate of glucose clearance either before or during insulin challenge (Figure 4c). An attractive interpretation of these data is that under insulin stimulation and PI3K activation, the protein product of the C‐allele suppresses the rate of peripheral glucose clearance, while the protein product of the T‐allele has no effect under these conditions. From these analyses, we concluded that the rs9400239 FOXO3A‐S longevity genotype is positively associated with peripheral insulin‐stimulated glucose clearance but does not influence the mRNA expression of either FOXO3A or FOXO3A‐S in the skeletal muscle.

### 2.4 FOXO3A‐S suppresses glycolytic flux in myotubes with PI3K‐dependency altered by the rs9400239 genotype

**FIGURE 5:**
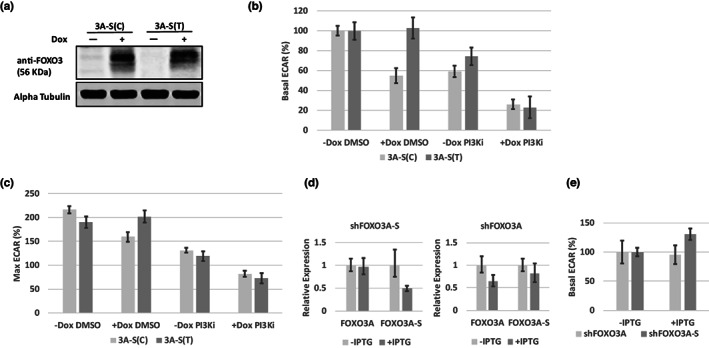
FOXO3A‐S suppresses glycolysis in myotubes with PI3K‐dependency altered by the rs9400239 genotype. (a) Western blot of the inducible overexpression of 3A‐S(C) or 3A‐S(T) constructs in LHCNM2 myotubes. Dox (0.1 μg/ml) was applied on Day 5 of differentiation, and on Day 7, the myotube lysates were harvested. (b) Seahorse assays performed following the same differentiation and Dox treatment as in (a) with the addition of DMSO or 5 μM GDC‐0941 (PI3Ki) for 1 h prior to the assay. All basal ECAR values were calculated as a percentage of the −Dox/DMSO control for each respective assay. The presented assay is representative of three independent replicates. Error bars are SEM. (c) Comparison of the maximal ECAR values upon oligomycin injection attained from the same overexpression assays performed in (b). The max ECAR was calculated as a percentage of the −Dox/DMSO basal ECAR within each assay. Error bars are SEM. (d) RT‐qPCR for inducible shRNA‐mediated knockdown of FOXO3A and FOXO3A‐S. Myoblasts were differentiated to myotubes and on Day 2 2 mM IPTG was applied with RNA harvested on Day 6. Both FOXO3A and FOXO3A‐S expression in the +IPTG condition was quantified relative to the −IPTG control. Error bars are SD. (e) ECAR measurement in myotubes where FOXO3A and FOXO3A‐S was inducibly knocked down (IPTG Day 2 measured Day 6). Basal ECAR values were calculated as a percentage of the −IPTG control. The presented assay is representative of three independent replicates. Error bars are SEM.

From the cohort results, we hypothesized that each of the FOXO3A‐S variants might have a differential effect on muscle‐mediated glucose clearance, with this difference possibly being insulin/PI3K‐dependent. To begin evaluating the role of the FOXO3A‐S transcripts in the regulation of glucose clearance, we chose to measure the extracellular acidification rate (ECAR, a measure of glycolytic flux) in LHCNM2‐derived myotubes. We constructed doxycycline‐inducible overexpression lines with the 3A‐S(C) and 3A‐S(T) clones in a FOXO3A and FOXO3A‐S wild‐type background (Figure 5a). We observed no perturbation of PI3K signaling when either of these lines was induced by Dox and observed complete inhibition of PI3K signaling with GDC‐0941 treatment (Figure S3). Overexpression of the 3A‐S(C) clone in myotubes resulted in repression of basal glycolytic flux (Figure 5b; −Dox/DMSO vs. +Dox/DMSO *p* < 0.0001). Overexpression of 3A‐S(C) also enhanced the effect of PI3K inhibition causing maximal repression of basal glycolytic flux (Figure 5b; −Dox/PI3Ki vs. +Dox/PI3Ki *p* = 0.0055). Overexpression of the 3A‐S(T) clone did not repress basal glycolytic flux on its own (Figure 5b). However, 3A‐S(T) overexpression enhanced the repressive effect on basal glycolytic flux caused by inhibition of PI3K (Figure 5b; −Dox/PI3Ki vs. +Dox/PI3Ki, *p* = 0.0031). We also assessed glycolytic capacity in these same assays following oligomycin injection. Overexpression of 3A‐S(C) alone repressed glycolytic capacity (Figure 5c; −Dox/DMSO vs. +Dox/DMSO, *p* < 0.0001) and enhanced the repressive effect of PI3K inhibition on glycolytic capacity (Figure 5c; −Dox/PI3Ki vs. +Dox/PI3Ki, *p* = 0.0007). 3A‐S(T) alone had no effect on glycolytic capacity, while it did enhance the repressive effect of PI3K inhibition on glycolytic capacity (Figure 5c; −Dox/PI3Ki vs. +Dox/PI3Ki, *p* = 0.01). These assays indicated that the C‐allele was capable of repressing glycolysis independently of PI3K signaling, while the T‐allele only repressed glycolysis when PI3K signaling was inactivated.

To further confirm FOXO3A‐S as a glycolytic repressor, we constructed IPTG‐inducible shRNA knockdown lines of FOXO3A and FOXO3A‐S (Figure 5d). Importantly, LHCNM2 is homozygous for the C‐allele of FOXO3A‐S as it is the only detectable form of FOXO3A‐S on the mRNA level (Figure S2f). Knockdown of FOXO3A in LHCNM2 myotubes had no effect on basal glycolytic flux (Figure 5e). A 50% knockdown of FOXO3A‐S caused a 30% increase in basal glycolytic flux (Figure 5e; *p* = 0.022). This confirmed that the C‐allele of FOXO3A‐S is a repressor of glycolytic flux in myotubes. Collectively, these results demonstrate that the alleles are functionally different in the regulation of glycolysis and that this difference is in accordance with the in vivo genotype association: the T‐allele may allow for higher insulin‐stimulated glucose clearance levels in vivo due to its inability to inhibit glycolysis under insulin‐stimulated (PI3K active) conditions. In contrast, the C‐allele is capable of glycolytic repression even in the presence of insulin/PI3K signaling; explaining the inverse correlation between C‐allele transcript levels and insulin‐stimulated peripheral glucose clearance.

### 2.5 FOXO3A‐S subcellular localization is unaltered by insulin/PI3K signaling in myotubes

Since FOXO3A cytosolic/nuclear localization is known to be regulated by PI3K/Akt signaling (Greer & Brunet, 2005), we checked to see where the FOXO3A‐S proteins localize in myotubes when the PI3K pathway is manipulated. We hypothesized this could explain the conditional repression of glycolytic flux by 3A‐S(T). To work in a clean background, we again utilized our 3A/3A‐S KO cells that were reconstituted with the 3A‐S(C) and 3A‐S(T) constructs for immunofluorescence (IF). We first tested IF staining with a c‐terminal FOXO3A antibody in LHCNM2 NT and 3A/3A‐S KO myotubes to validate that there was low background staining under either insulin or PI3K‐inhibited (GDC‐0941 treated) conditions (Figure S4). We then stained 3A/3A‐S KO myotubes induced to overexpress either canonical FOXO3A, 3A‐S(C), or 3A‐S(T) under these conditions. As expected, FOXO3A was restricted from the nucleus under insulin and shuttled into the nucleus with PI3K inhibition (Figure S5). In contrast, the FOXO3A‐S proteins exhibited both cytosolic and nuclear staining that was unaltered by PI3K pathway manipulation (Figure S5). This led us to rule out subcellular localization as the PI3K‐mediated regulatory mechanism modulating conditional 3A‐S(T) glycolytic flux suppression.

## 3 DISCUSSION

**FIGURE 6:**
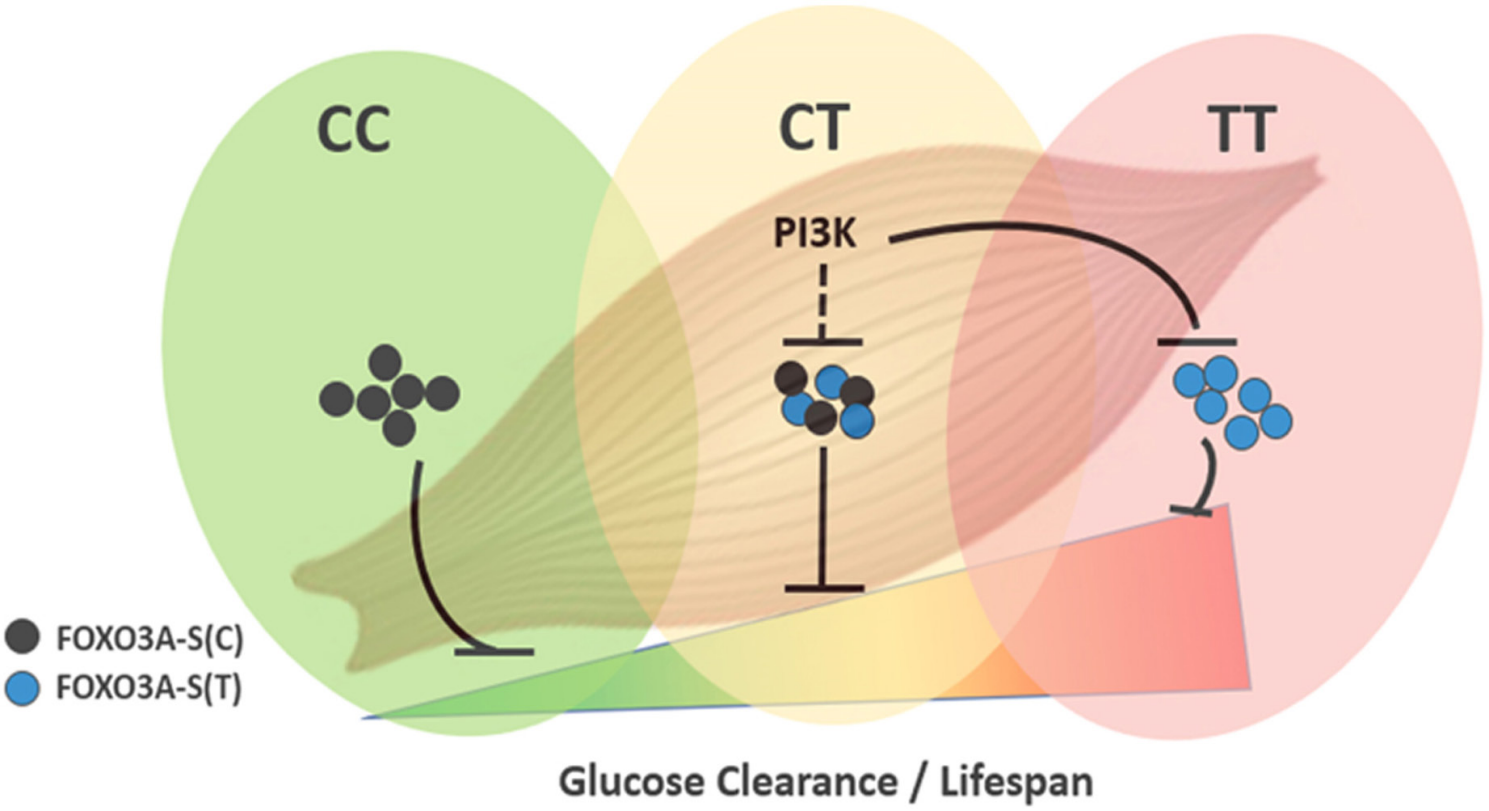
Model of FOXO3A‐S action in longevity and peripheral glucose clearance. The FOXO3A‐S proteoforms either constitutively or conditionally limit skeletal muscle‐mediated glucose clearance under insulin‐stimulated conditions. Longevity (T‐allele) carriers express a PI3K‐repressible form of the protein allowing for higher glucose clearance rates with insulin challenge while C‐allele carriers have more restricted insulin‐stimulated glucose clearance because the C‐allele proteoform is refractory to PI3K signaling. Increased peripheral glucose clearance confers health benefits to T‐allele carriers, which allows for longer and healthier lives.

Our renewed genetic analysis of the FOXO3A locus has identified FOXO3A‐Short and its regulatory polymorphism rs9400239 as novel mediators of the effect of the FOXO3A genotype on both insulin sensitivity and presumably human longevity. Given the coincident genetic mapping of these phenotypes within the FOXO3A locus, it seems likely that enhanced peripheral insulin‐stimulated glucose clearance may be strongly contributing to extraordinary human lifespan extension. Our model posits that due to the ability of PI3K signaling to inactivate the protein product of the longevity T‐allele and the inability of PI3K to inactivate that of the non‐longevity C‐allele glucose clearance is enhanced in longevity allele carriers during insulin challenge (Figure 6). Furthermore, the highly restricted expression pattern of FOXO3A‐S suggests that the skeletal muscle is the primary mediator of these phenotypes, implicating the skeletal muscle as a particularly important tissue modulating human healthspan and lifespan. Indeed, increased muscle mass is positively correlated with insulin sensitivity and inversely correlated with all‐cause mortality in humans (Srikanthan & Karlamangla, 2011, 2014).

Our model that enhanced peripheral glucose clearance is positively associated with longevity in humans is supported by other human studies as well as data from primates and model organisms. The Leiden Longevity Study found that peripheral glucose clearance is positively associated with familial longevity, while hepatic glucose clearance was not (Wijsman et al., 2011). Additionally, a low insulin resistance index (HOMA‐IR) among centenarians has been observed when compared to their younger controls (Paolisso et al., 2001). In mouse models, both caloric restriction as well as growth hormone receptor knockout (GHRKO) caused increased lifespan and insulin sensitivity (Masternak et al., 2009). GHRKO mice with genetically normalized insulin sensitivity exhibited reversion of GHRKO benefits in various age‐related parameters, demonstrating a causative role for insulin sensitivity in the modulation of aging phenotypes (Arum et al., 2014). Corroborating this finding, total ablation of the insulin receptor in all the peripheral tissues of adult mice shortened lifespan (Merry et al., 2017). In monkeys, a causative association between caloric restriction, increased insulin‐stimulated PI3K activity in skeletal muscle, increased insulin sensitivity, and enhanced peripheral glucose clearance has also been observed (Wang et al., 2009). In a separate cohort of monkeys, caloric restriction was shown to significantly increase lifespan (Colman et al., 2014); this was also confirmed in gray mouse lemurs (Pifferi et al., 2018). In humans, caloric restriction was found to have no effect on insulin‐stimulated oxidative glucose metabolism in skeletal muscle while enhancing glucose disposal by increasing non‐oxidative glucose metabolism (Johnson et al., 2016). Furthermore, increased non‐oxidative glucose metabolism concurrent with strongly suppressed oxidative glucose metabolism in the skeletal muscle has been found to increase skeletal muscle glucose disposal and reduce age‐ and diet‐dependent adiposity in mice (Sharma et al., 2019). The diabetes medication metformin also increases non‐oxidative glucose metabolism in human skeletal muscle while having no or perhaps even a negative effect on oxidative glucose metabolism (Musi et al., 2002). Importantly, metformin reduces all‐cause mortality in both healthy and diabetic humans, making it a general geroprotective compound (Campbell et al., 2017). Interestingly, oxygen consumption rate (OCR) which was measured simultaneously with ECAR on the seahorse was not significantly altered by FOXO3A‐S overexpression, either in combination with PI3K inhibition or alone (data not shown). This strongly suggests that FOXO3A‐S primarily regulates non‐oxidative glucose metabolism, pinpointing this pathway as being of specific importance to human longevity. Furthermore, this mechanism may be conserved as recent work on fruit flies demonstrates that specific genetic enhancement of glycolysis was found to extend lifespan (Ma et al., 2018). It is also notable that mitochondrial function and OXPHOS complexes decline with age in the skeletal muscle of primates and humans, but not in rats (Mercken et al., 2017). This age‐dependent decline in skeletal muscle oxidative capacity may render skeletal muscle non‐oxidative glucose metabolism increasingly crucial for the maintenance of whole‐body glucose homeostasis in primates and humans as they age, paralleling the evolution of FOXO3A‐S as a novel regulator of this physiology. Taken together, our findings that the non‐longevity C‐allele suppresses glycolytic flux in skeletal muscle independently of insulin/PI3K signaling while the longevity‐conferring T‐allele is suppressed by insulin/PI3K signaling allowing for maximal glycolytic flux is highly consistent with these previous reports.

The PI3K/Akt pathway is of central importance in human glycemic regulation (George et al., 2004). Mechanistically, our findings suggest that FOXO3A‐S may be acting as another arm of insulin signaling that both interacts with and parallels PI3K signaling in the regulation of glycolysis in humans. Both alleles of FOXO3A‐S encode a protein capable of suppressing glycolysis additively with PI3K inhibition, which on its own completely inhibited both Akt and mTORC1 signaling in these myotubes (Figure S3). This observation strongly suggests that FOXO3A‐S regulates aspects of glycolysis that the PI3K/Akt/mTORC1 pathway does not control. Indeed, the potency of these FOXO3A‐S proteoforms in glycolytic regulation may explain why they exhibit exceptionally tight transcriptional, translational, and post‐translational regulatory mechanisms. In the future, it will be of great interest to discern exactly how FOXO3A‐S exerts its effects on glycolysis and how the T‐allele proteoform is conditionally regulated by insulin/PI3K signaling.

Our findings also reinforce the tissue specificity by which insulin signaling influences lifespan in mammals. The SNP and PI3K‐dependent differences in FOXO3A‐S functionality argue that increased insulin signaling in skeletal muscle is specifically beneficial for longevity in humans; otherwise no glucose metabolism and presumably longevity advantage would accrue to T‐allele carriers. This conclusion extends previous findings that increased insulin signaling in adipose tissue and decreased insulin signaling in all peripheral tissues are both situations detrimental to longevity (Bluher et al., 2003; Merry et al., 2017). Last, our results implicate FOXO3A‐S as essentially an anti‐longevity molecule, fundamentally challenging the prevailing view that increased FOXO activity will almost always be geroprotective. This makes the minimally conserved FOXO3A‐S isoform a cautionary tale in the translation of concepts from model organisms to humans, highlighting how rapid evolution in a single family member can upset a paradigm. In the future, deeper study of FOXO3A‐S will be of great utility in the elucidation of new genes and pathways directly influencing glucose metabolism and human longevity.

## 4 EXPERIMENTAL PROCEDURES

### 4.1 Danish twin cohort

The cohort used here has been previously described and characterized (Banasik et al., 2011). The twin population included 196 monozygotic (MZ; *n* = 108) and same‐sex dizygotic (DZ; *n* = 88) Danish twins without known type 2 diabetes. The population consisted of two age groups, young (28 ± 2 year, *n* = 110) and elderly (62 ± 2 year, *n* = 86) participants. All participants were Danes by self‐report, and written informed consent was obtained from all individuals before participation. There was no significant difference in glucose tolerance status between MZ and DZ twins within each age group. Zygosity was determined by polymorphic genetic markers. The study was approved by the regional ethical committees, and the study was conducted according to the principles of the Helsinki Declaration.

### 4.2 Cell lines, culture conditions & reagents

All lines were cultured on Greiner BioOne (CELLSTAR) plasticware – this was especially relevant for LHCNM2 myoblasts. HEK293T cells were cultured in high‐glucose Dulbecco's Modified Eagle Medium (DMEM) (Gibco cat. #11965) supplemented with 10% fetal bovine serum, 20 mM L‐glutamine, 10 U/ml penicillin, and 10 μg/ml streptomycin and maintained at 37°C under 5% CO_2_. The immortalized human myoblast line LHCNM2 was previously described (Zhu et al., 2007). All cell lines were repeatedly tested for mycoplasma contamination by PCR and found to be mycoplasma‐free. These cells were cultured on collagen‐coated plates (plates pre‐incubated at 37°C for at least 1 h with a PBS solution of 10 μg/ml collagen from rat tail, Sigma) at 37°C under 5% CO_2_. The base of the growth medium was a 4:1 mixture of high‐glucose DMEM (Gibco cat. #11965) and Medium 199 (Gibco cat. #11150–059) buffered with bicarbonate. Additional to this was 0.02 M HEPES, 20% fetal bovine serum, 0.03 μg/ml zinc sulfate (Sigma), 1.4 μg/ml vitamin B12 (Sigma), 0.055 μg/ml dexamethasone (Sigma), 2.5 ng/ml human HGF (Peprotech), 10 ng/ml human basic FGF (Peprotech), 10 U/ml penicillin, and 10 μg/ml streptomycin. For differentiation, the cells were grown to maximal confluency, and then the medium was changed to serum‐free differentiation medium after washing 1x with PBS to remove residual growth medium. This medium contained 4:1 DMEM:M199, 0.02 M HEPES, 0.03 μg/ml zinc sulfate, 1.4 μg/ml vitamin B12, 10 μg/ml human insulin (Sigma), 10 U/ml penicillin, and 10 μg/ml streptomycin. Following the initial medium change, the cells were switched to 3% CO_2_ and then the medium was changed every 2 days with differentiation usually becoming apparent on Day 5 with maturation being reached on Days 6 & 7. The compounds GDC‐0941 and bortezomib were purchased from Selleckchem and dissolved to 5 mM and 1 mM stocks, respectively, in Hybri‐Max DMSO (Sigma). Doxycycline and cycloheximide were from Sigma. Blasticidin and puromycin were from Invitrogen.

### 4.3 Statistical analysis

Unpaired Student's *t* tests were used to evaluate statistical significance. Values are expressed as means ± SEM or means ± SD, as indicated. To assess correlations Pearson's correlation coefficients were calculated on normally distributed data. The Danish cohort data were analyzed using mixed‐ANOVA regressions correcting for appropriate parameters as described. All *p*‐values were calculated based on 95% confidence intervals. See figure legends for sample size and replicate details.

See Supporting Information for additional experimental procedures.

## AUTHOR CONTRIBUTIONS

Evan E. Santo discovered FOXO3A‐Short. Evan E. Santo, Jihye Paik, Ellen M. Westerhout, and Rogier Versteeg designed experiments. Allan A. Vaag provided the Danish cohort data. Rasmus Ribel‐Madsen, Evan E. Santo, and Allan A. Vaag analyzed the Danish cohort data. Evan E. Santo, Ellen M. Westerhout, Peter J. Stroeken, Ninna S. Hansen, and Maaike Commandeur performed experiments. Vincent C. J. de Boer aided with seahorse experiments. The manuscript was written by Evan E. Santo and edited by Rasmus Ribel‐Madsen, Allan A. Vaag, Jihye Paik, and Ellen M. Westerhout.

## CONFLICT OF INTEREST

The authors declare no competing interests.

## Supporting information


Appendix S1

## Data Availability

The full dataset (genotyping, RT‐qPCR and glucose clearance measurements) for the Danish cohort is available on request from the corresponding author E.E.S. The cDNA sequences for the full‐length FOXO3A‐Short clones have been deposited in GenBank under submission IDs MK390614 and MK390615. All constructs and cell lines used in this paper can be requested from E.E.S. and J.P.
