# Supplementary material for: FOXO3A‐short is a novel regulator of non‐oxidative glucose metabolism associated with human longevity
Source: Aging Cell. 2023 Jan 8;22(3):e13763. doi: 10.1111/acel.13763 (PMC10014046; doi:10.1111/acel.13763)
Supplement: Supplementary file 1 — Appendix S1 [file ACEL-22-e13763-s001.pdf]

FIGURE S1

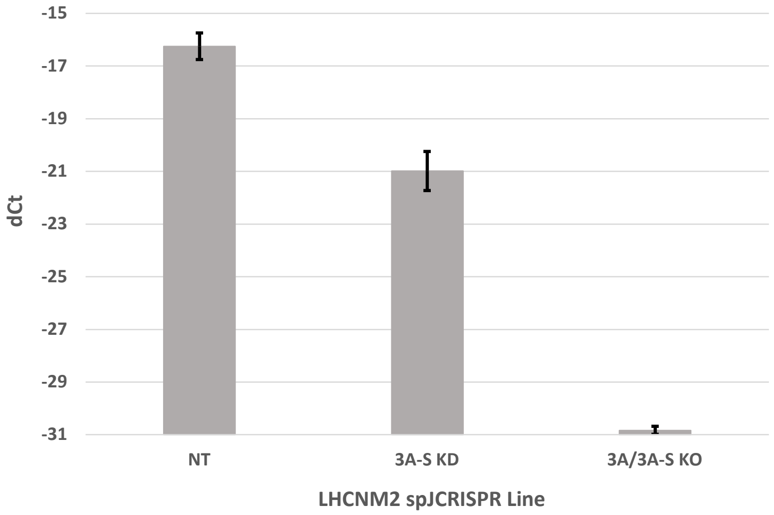

FIGURE S1 Knockdown of FOXO3A-S in LHCNM2 myotubes using spJCRISPR. LHCNM2 spJCRISPR lines were differentiated and harvested for total RNA on Day 7. RT-qPCR was performed for FOXO3A-S and 18S rRNA then the ΔCt was calculated. Error bars are SD of three replicates.

FIGURE S2

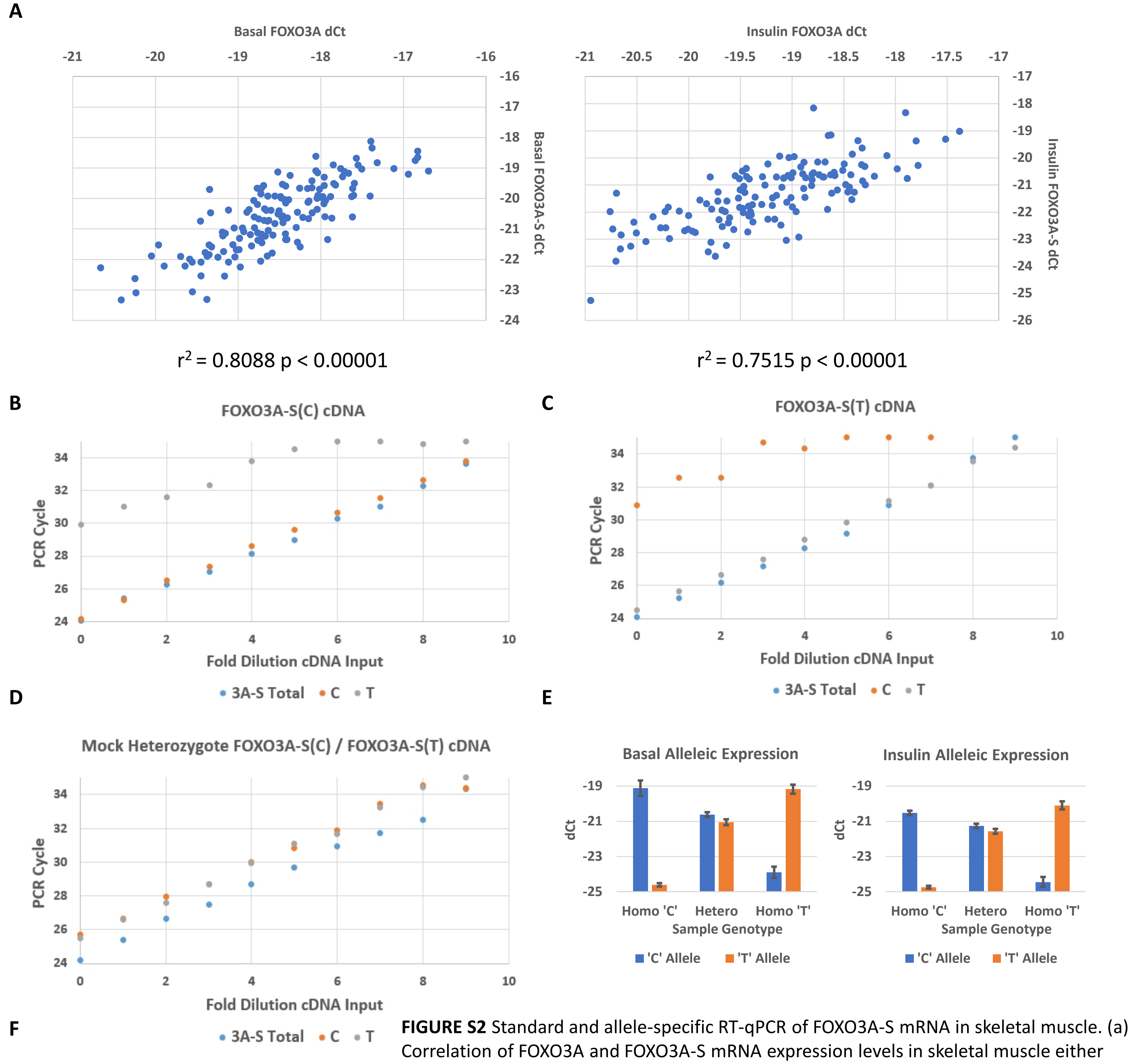

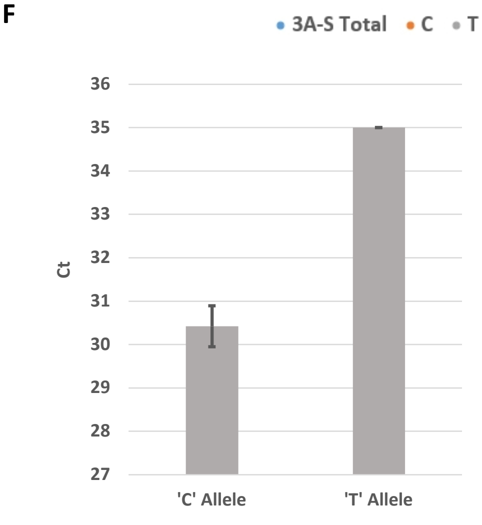

FIGURE S2 Standard and allele-specific RT-qPCR of FOXO3A-S mRNA in skeletal muscle. (a) Correlation of FOXO3A and FOXO3A-S mRNA expression levels in skeletal muscle either before or during insulin infusion. RT-qPCR was performed using conventional FOXO3A-S and FOXO3A primers; expression was calculated relative to 18S rRNA using the ΔCt method. Pearson correlation coefficients are shown. (b) qPCR of serial dilutions of cDNA made from muscle biopsy mRNA from an individual homozygous for the C-allele of FOXO3A-S. Three different forward primers were used in three separate parallel reactions in combination with the same reverse primer. "3A-S Total" uses the allele-insensitive standard FOXO3A-S RT-qPCR forward primer while 'C' is the forward specific for C-allele cDNA and 'T' is the forward specific for T-allele cDNA. (c) The same dilutions and measurements as in Figure S2B but with cDNA from an individual homozygous for the T-allele of FOXO3A-S. (d) qPCR of serial dilutions made from a mix of the cDNAs used in Figure S2A and Figure S2B. Prior to mixing the cDNAs each cDNA was individually adjusted to where a target Ct of approximately 24 was achieved for FOXO3A-S with the "3A-S Total" primer set. These cDNA dilutions were then mixed 1:1 to create a "mock heterozygote" where half the cDNA contains C-allele FOXO3A-S and the other half T-allele FOXO3A-S. qPCR was performed with all three primer sets on the dilution series made from this mix. (e) Allelic expression of FOXO3A-S from all muscle biopsy samples normalized to 18S rRNA using the ΔCt method. Genotypes on the x-axis were determined by genotyping genomic DNA. 'C' Allele and 'T' Allele denote the allele-specific RT-qPCR primer pairs used. Error bars are SD. (f) Allele-specific RT-qPCR for FOXO3A-S in cDNA synthesized from LHCNM2 myotube mRNA. 'C' Allele and 'T' Allele denote the primer sets used. Error bars are SD of four replicates.

FIGURE S3

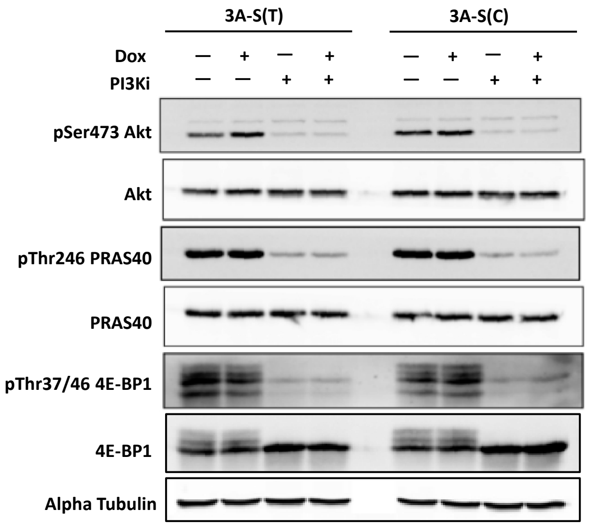

FIGURE S3 PI3K pathway inhibition and activity in LHCNM2 myotubes. Western blot of PI3K/Akt/mTOR pathway activity readout phosphorylation sites in LHCNM2 myotubes (FOXO3A and FOXO3A-S wildtype background) overexpressing either the 3A-S(C) or 3A-S(T) constructs. Dox (0.1 µg/ml) was applied or not on Day 5 of differentiation and on Day 7 the myotubes were treated with DMSO or 5 µM GDC-0941 (PI3Ki) for 1 hour then harvested.

FIGURE S4

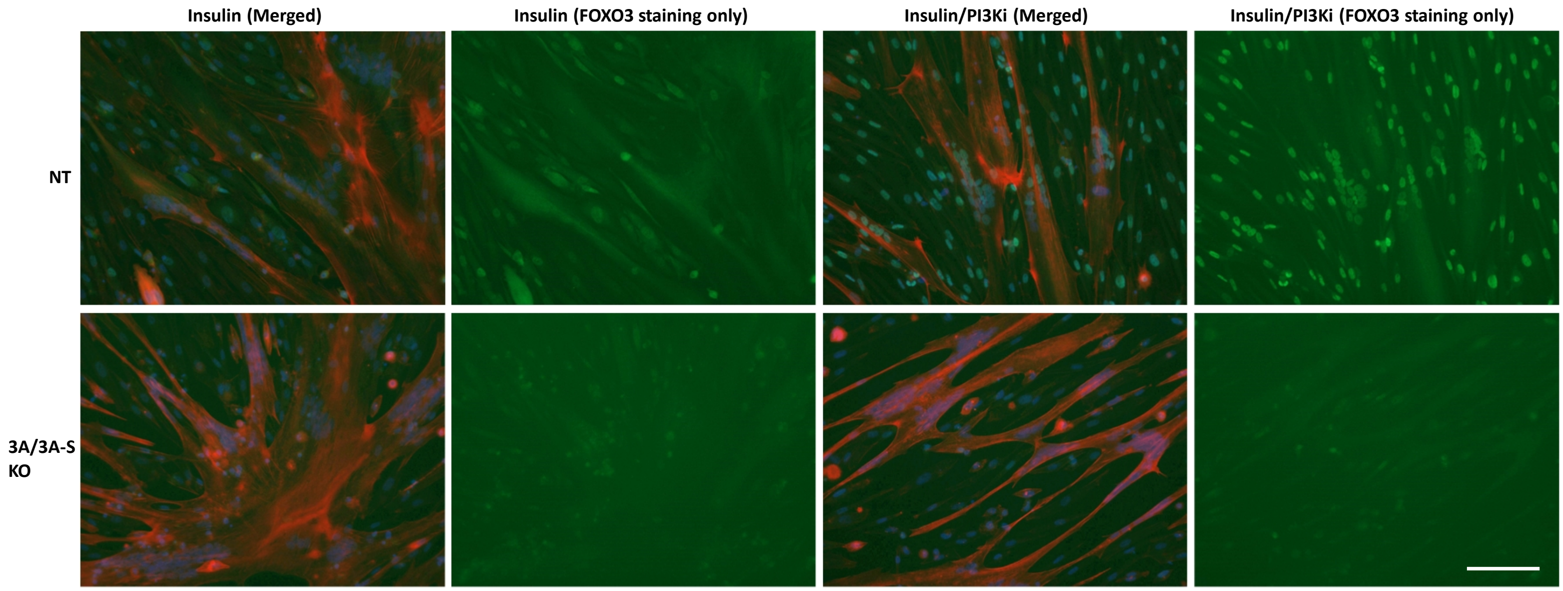

FIGURE S4 Immunofluorescence of endogenous FOXO3A in LHCNM2 myotubes. LHCNM2 spJCRISPR lines were differentiated then on Day 7 the cultures were either treated with 200 nM insulin or 200 nM insulin + 5 µM GDC-0941 (PI3Ki) for 1 hour followed by fixation and staining with DAPI (blue), phalloidin (red) and FOXO3A c-terminal antibody (green). The white scale bar is 25 µm.

FIGURE S5

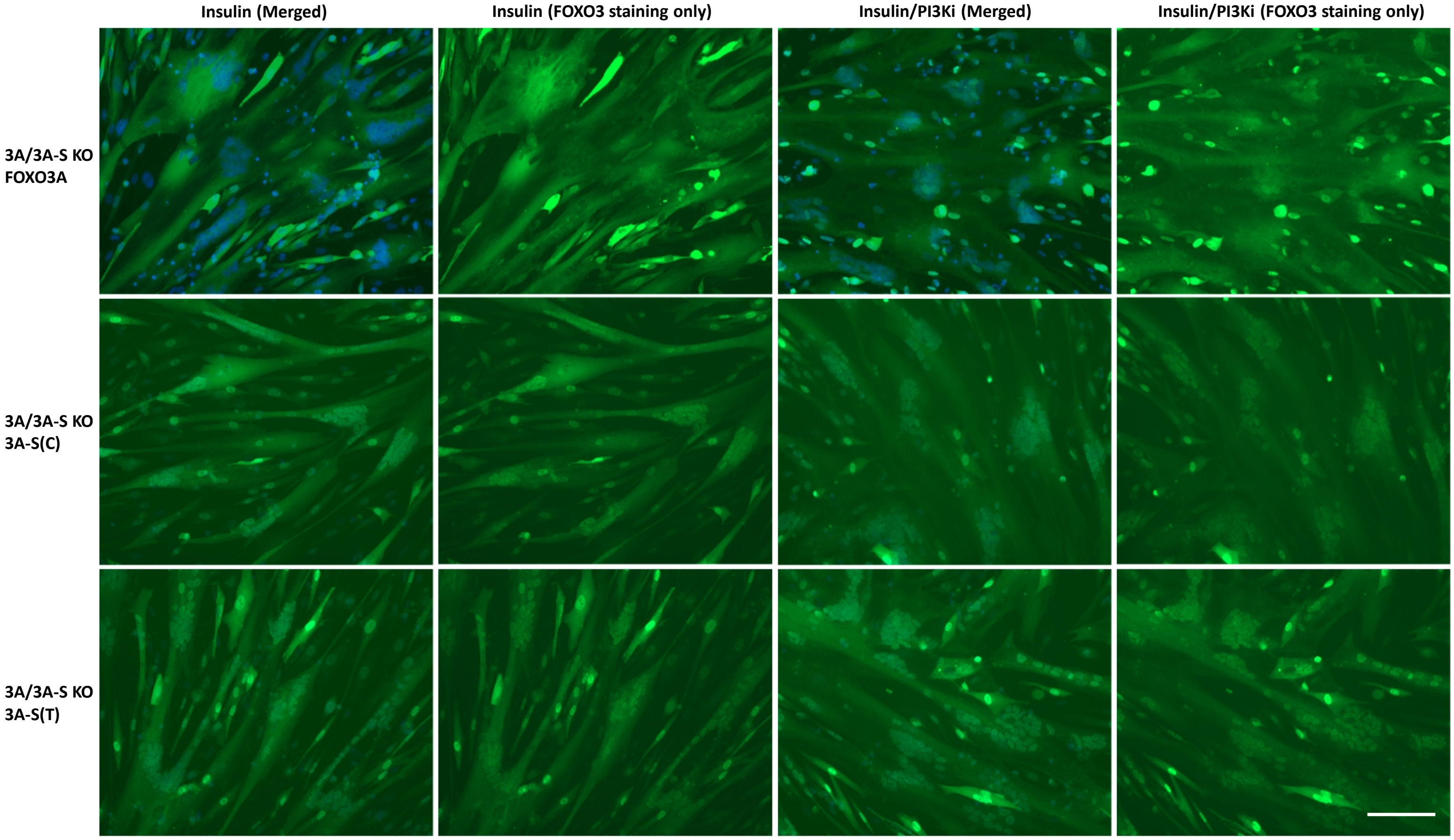

FIGURE S5 FOXO3A-S proteoform subcellular localization is not affected by insulin/PI3K/Akt signaling. Immunofluorescence images of myotubes derived from LHCNM2 3A/3A-S KO cells transduced with Dox-inducible overexpression constructs of FOXO3A, 3A-S(C) or 3A-S(T). Dox was added on Day 5 of differentiation, on Day 7 the cultures were either treated with 200 nM insulin or 200 nM insulin + 5 µM GDC-0941 (PI3Ki) for 1 hour followed by fixation and staining with DAPI (blue) and FOXO3A c-terminal antibody (green). The white scale bar is 25 µm.

TABLE S1

No-Target_1_Fwd	**ACCG**CTCCGTTATGTGGCATGAGA
No-Target_1_Rev	**AAAC**TCTCATGCCACATAACGGAG
No-Target_2_Fwd	**ACCG**GTAAAGAAGCGGAAAGGTCC
No-Target_2_Rev	**AAAC**GGACCTTTCCGCTTCTTTAC
FOXO3A-S_TS_MULE_Fwd	**ACCG**GTTCCAGGGCGGCCCCTTCC
FOXO3A-S_TS_MULE_Rev	**AAAC**GGAAGGGGCCGCCCTGGAAC
FOXO3A-S_BS_MULE_Fwd	**ACCG**GGATGTCACTGCTTCCGTGT
FOXO3A-S_BS_MULE_Rev	**AAAC**ACACGGAAGCAGTGACATCC
FOXO3A-3A-S_TS_MULE_Fwd	**ACCG**GTGCCGGATGGAGTTCTGCA
FOXO3A-3A-S_TS_MULE_Rev	**AAAC**TGCAGAACTCCATCCGGCAC
FOXO3A-3A-S_BS_MULE_Fwd	**ACCG**GGGGTCCAGAATGAGGGAAC
FOXO3A-3A-S_BS_MULE_Rev	**AAAC**GTTCCCTCATTCTGGACCCC

TABLE S1 spJCRISPR sgRNAs. Listed here are the full oligos used to clone the sgRNAs inclusive of the BfuAI overhangs in bold. Top and bottom strand-targeting sgRNAs within each pair are denoted by 'TS' and 'BS', respectively. No-Target (NT) sgRNAs are lacking a PAM sequence.

SUPPLEMENTAL EXPERIMENTAL PROCEDURES

Modification of pInducer20 Lentiviral Destination Vector

The pInducer20 vector was modified to contain a blasticidin resistance gene In place of the neomycin resistance cassette (Meerbrey et al., 2011). This was done by first re-cloning a portion of the TetR-IRES-NeoR cassette from the pInducer20 vector into our pENTR-MCS vector (Santo et al., 2012). The primers used were:

5'-gatcatactagtcatatgtggcctggagaaacag-3' (fwd)

5'-gatcatctcgaggcatgctcgccttgagcctggc-3' (rev)

The PCR product was digested with SpeI (5') and XhoI (3') and ligated into pENTR-MCS. The BlaR gene was amplified from the pLenti6.3 CMV/TO vector (Invitrogen) with the primers:

5'-gatcataccggtatggccaagcctttgtctcaag-3' (fwd)

5'-gatcatgcatgcttagccctcccacacataaccag-3' (rev).

This product and the pENTR-TetR-IRES-NeoR were digested on AgeI (5') and SphI (3') and ligated, destroying the NeoR cassette. This new product and the pInducer20 vector were then digested on NdeI (5') and SphI (3') and ligated creating pInducer-BlaR. Subclones were sequence verified and screened for unwanted recombination by digesting with ScaI and SphI.

5’ RACE Of FOXO3A-Short

To map the 5' end of FOXO3A-Short we performed 5' RACE with the Invitrogen RACE kit (cat. #15590-101 & 18374-041) using RNA from human LHCNM2 myotubes. The cDNA was first synthesized with a reverse primer to exon 3 of FOXO3A 5'-gctggcgttagaatt-3' Following RNA degradation this cDNA was then purified using the provided S.N.A.P. columns and tailed as described in the kit. The initial PCR was performed with the kit provided abridged anchor primer and the reverse primer 5'-ctgccaggccacttggagag-3' (RACE2). Nested PCR was then performed with the AUAP kit primer and the reverse primer 5'-gcgcggccacggctctt-3' (RACE1). The PCR products of this reaction were then TOPO cloned (Invitrogen cat. #45-0641) and the clones were sequenced with the kit provided M13 forward and reverse primers.

Generation Of FOXO3A & FOXO3A-Short Overexpression Clones

FOXO3A was PCR amplified from our previous pENTR-MCS-HA-FOXO3A clone (Santo et al., 2013). This was done to remove the HA tag and re-cloned into pENTR-MCS on HindIII (5’) and BamHI (3’), creating a wild-type clone of FOXO3A. Using the sequence information from the 5’ RACE the cloning primer 5’-gatcataagcttaaaaacctctctgtgttccaggggaagcacatgcagctgg-3' (fwd) was used in combination with the RACE2 primer (rev) to amplify the 5’ end of FOXO3A-Short. The RACE2 primer captures an NcoI site present within exon 3 of FOXO3A (exon 2 of FOXO3A-Short). This product and the pENTR-MCS-FOXO3A clone were digested on HindIII (5’) and NcoI (3’) and ligated generating a wild-type clone of FOXO3A-Short. Since the source material was LHCNM2 which is homozygous for the C-allele of the rs9400239 SNP we subsequently used the QuikChange II Site Directed Mutagenesis Kit (Stratagene) to generate a subclone with the T-allele variant. These two clones are referred to as 3A-S(C) & 3A-S(T). We also generated a clone where expression was enforced from the first ATG in-frame with the canonical FOXO3A stop codon. To generate the 3A-S(ATG) clone we used the primers:

5’-gatcataagcttgccaccatgcgggtccagaatgagggaac-3’ (fwd)

5’-ctattgtccatggagacagcc-3’ (rev)

This fragment was cloned back into the pENTR-MCS-3A-S(C) clone by digestion on HindIII (5’) and NcoI (3’); removing the 5’ UTR. These pENTR clones were then used in Clonase LR II (Invitrogen) reactions with either the pCDNA-DEST40 (Invitrogen) vector or the pInducer-BlaR vector. All clones were thoroughly sequenced to ensure their integrity and identity in both the pENTR and destination vector backbones.

Construction Of Inducible shRNA Knockdown Clones

To target FOXO3A specifically we used our previous pLKO.1 3xLacO shFOXO3A construct which contains an IPTG inducible shRNA against exon 2 of FOXO3A (Santo et al., 2013). In place of this shRNA we cloned a shRNA specific for exon 1 of FOXO3A-Short. We digested the shFOXO3A vector on Acc65I (5') and EcoRI (3') and ligated the annealed sequences:

5'-GTACC**GAAGCGGACGTAGGGTACAAA**CTCGAG**TTTGTACCCTACGTCCGCTTC**TTTTTG-3' (fwd) and 5'-AATTCAAAAA**GAAGCGGACGTAGGGTACAAA**CTCGAG**TTTGTACCCTACGTCCGCTTC**G-3' (rev) where the targeting sequence is in bold and the loop and transcriptional terminator are underlined.

Construction Of spJCRISPR Clones

The spJCRISPR constructs were cloned as previously described (Santo & Paik 2018) using the pairs of sgRNAs described in Table S1.

Lentiviral Packaging & LHCNM2 Line Construction

Viruses were packaged as described previously for construction of LHCNM2 FOXO3A-Short Dox-inducible overexpression lines in a WT FOXO3A/3A-S background and the inducible shRNA lines (Santo et al., 2013). Viral titers were quantified by p24 ELISA. LHCNM2 myoblasts were transduced for 48 hours with a titration of each construct based on p24 values in a 6-well plate format. This was calculated as nanograms of virus per 100,000 cells for each construct. Cells were then selected with either 10 µg/ml blasticidin (pInducer-BlaR) or 6 µg/ml puromycin (pLKO.1 3xLacO). For blasticidin to have an effect these cells required one passage, this was not the case for puromycin. To estimate an MOI of 1 for each construct points in each titration were chosen where most cells exhibited near complete drug resistance relative to unselected controls. Higher MOIs were then calculated based on the ng/100,000 cells where an MOI of 1 was observed. Typically lines could be established at an MOI of 3 with no discernable proliferation, morphological or differentiation defects. For the 3xLacO shRNA constructs a super-transduction was performed based on the MOI estimates obtained from the initial transduction. For the spJCRISPR constructs and spJCRISPR FOXO3A/3A-S knockout clone transduced with inducible overexpression constructs virus was produced and infections were performed as previously described (Santo & Paik, 2018). LHCNM2 cells transduced with FOXO3A/3A-S and FOXO3A-Short-specific spJCRISPR constructs were single-cell FACS sorted to 96-well plates for clonal isolation. All lines were allowed to fully recover prior to experiments.

RT-qPCR & Conservation RT-PCR

Total RNA was harvested using Trizol (Life Technologies) and purified according to standard protocols. The RNA for the panel of human tissues was purchased from Clontech (cat. #636643). The RNA from the human muscle biopsies before and after oral glucose challenge was previously isolated (Banasik et al., 2011). cDNA was synthesized using 1 µg of RNA as input in a SuperScript III reaction using random hexamers (Life Technologies). cDNA was diluted 1:10 from the original synthesis reaction volume prior to qPCR. qPCR was performed for each cDNA in quadruplo (1 µl cDNA per reaction) using PowerUp SYBR Green Master Mix (Thermo A25741) and the Roche LightCycler 480 384-well platform with an annealing temperature of 56°C and a standard program of 40 cycles. Absolute quantitation was used in the analysis software to obtain the Ct values. To measure FOXO3A and FOXO3A-Short specifically the same reverse primer 5'-cattctggacccgcatgaatcg-3' (FOXO3A exon 3) was used with specific forward primers for FOXO3A (5'-gtgcgttgcgtgccctacttc-3') and FOXO3A-Short (5'-gttacttctgactggcacgg-3'). These primers were used throughout this study but we did find it necessary to design different forward primers in order to measure shRNA knockdown by RT-qPCR. Specifically, we had to position the shRNA target sites for both FOXO3A and FOXO3A-Short to be within the PCR product in order to quantitate the effect of the shRNAs. This phenomena has been described previously (Holmes, Williams, Chapman, & Cross, 2010). The shRNA knockdown forward primers were 5'-catcgagagctccccggacaaa-3' (FOXO3A) and 5'-gacagtctccacaaggtaaacatgg-3' (FOXO3A-Short) and again used with the same reverse primer. The muscle differentiation marker MYH8 was measured with 5'-tgcaacaggagatttctgac-3' (fwd) and 5'-cagcctgaatttcacatttc-3' (rev). To probe for FOXO3A-Short conservation primers were designed to conserved sequence targeting FOXO3A-Short exon 1 (5'- ccacaaggtaaacatgg-3') and FOXO3A exon 3 (5'- caggttgtgccggatggagtt-3'). For the conservation PCR an annealing temperature of 52°C and 35 cycles was used. In all cases 18S rRNA was used as a reference 5'-ccgataacgaacgagactctgg-3' (fwd) and 5'-tagggtaggcacacgctgagcc-3' (rev). All PCR products from all primer sets were gel purified and sequenced to ensure the primers were specific and that no other products were being generated.

Allele-Specific RT-qPCR

The protocol, PCR program and machine were exactly the same for the allele-specific RT-qPCR as for the standard RT-qPCR except that the annealing temperature was 62°C. We tested multiple annealing temperatures and optimized for selectivity and sensitivity by looking for template cross-reactivity as well as gauging sensitivity using the standard FOXO3A-Short RT-qPCR primers described above. The C-allele forward was 5'-cggcacctctgaaatact**c**tt-3' and the T-allele forward was 5'-cggcacctctgaaatact**t**tt-3' and both were used

with the same FOXO3A-Short reverse primer described above. The site of the rs9400239 polymorphism is in bold. We tested other forward primers for selectivity and sensitivity with the rs9400239 SNP placed in various positions and found that selectivity was best maintained on the 3' end.

Genotyping

The Danish twin cohort (Banasik et al., 2011) was genotyped for rs9400239 using the KASP method (LCG Genomics, Herts, UK) with a success rate of 96%. Allele frequencies were in accordance with HapMap (minor allele frequency 29%, CEU population) and obeyed Hardy-Weinberg equilibrium ($p = 0.2$).

In Vitro Transcription/Translation Assay

The pCDNA-DEST40 vector has a T7 promoter in addition to the CMV, making it directly compatible with the rabbit reticulocyte-based TNT Quick Coupled Transcription/Translation kit (Promega). Reactions were carried out according to the standard protocol.

HEK293T Transfections

For all pCDNA-DEST40 derived clones used in transfection the plasmid DNA was carefully quantitated by Nanodrop in order to transfect equal amounts of all clones. HEK293T cells were seeded in 6 cm plates and grown to 90% confluency. For each transfection 10 µg of plasmid DNA was mixed with Fugene HD (Promega) in 500 µl of optimum in a 5:2 ratio (5 µl of Fugene HD : 2 µg of DNA). After a 15 minute incubation 5 ml of medium was added to the mix and the medium of the cells was replaced with this transfection medium. The next day cells were harvested for protein.

Antibodies

We raised rabbit polyclonal antibodies to a c-terminal peptide sequence of FOXO3A (Cys-MMTQSDPLMSQASTAVSAQN) which was conjugated to KLH then injected into two rabbits with subsequent 7-8 re-immunizations; each one done at 1-2 week intervals. After the terminal bleed antibodies were affinity purified using the peptide fixed to a column (GL Biochem(Shanghai) Ltd.). Antibodies were resuspended in 50% glycerol/dH_2O to a concentration of 1 mg/ml and stored at -20°C. Commercial antibodies used were all from Cell Signaling: FOXO3A (c-terminal #12829 1:1000) alpha tubulin (#3873 1:100,000), nuclear lamin A/C (#4777 1:50,000), pSer473 Akt (#4060 1:1000) pan-Akt (#4691 1:50,000), pThr246 PRAS40 (#2997 1:5000), PRAS40 (#2691 1:20,000), pThr37/46 4E-BP1 (#2855 1:1000), 4E-BP1 (#9644 1:10,000).

Western Blotting

For total extraction of FOXO3A-Short protein from all cellular compartments we found it necessary to use high SDS for lysis. Protein lysates were harvested in 4% SDS lysis buffer (4% SDS , 50 mM Tris-HCl pH 7.4) containing protease and phosphatase inhibitors, sheared with a 23 gauge needle, centrifuged at 13,000 rpm for 5 minutes then quantitated with the DC Protein Assay kit (Biorad 5000116). Lysates were then diluted to appropriate concentrations in 6x sample buffer, boiled for 10 minutes and resolved on 8% SDS-PAGE gels. To visual FOXO3A-Short from overexpression lysates 25 µg of protein per well would be loaded on 15-well gels. Gels were typically run until the 46 KDa marker was near the bottom to maximize FOXO3A-Short band resolution. Gels were wet transferred (20% methanol) to Amersham Protran Premium 0.45 um NC membrane (GE Healthcare 10600003) at 100 volts and 4°C for two hours. After Ponceau S staining membranes were blocked in 5% Milk TBS at RT for one hour then incubated with primary antibodies at appropriate dilutions in 3% BSA TBS + 0.1% Tween 20 (TBST) overnight rocking at 4°C. All FOXO3A / FOXO3A-Short westerns were incubated with 1:1000 dilution of Cell Signal FOXO3A #12829. After the primary membranes were washed three times with TBST and incubated with appropriate HRP-conjugated secondary antibody diluted in 5% Milk TBST for one hour at RT. ECL and film was used for signal detection. Western blot bands were quantitated using Image Studio Lite version 5.2 (LI-COR).

Immunoprecipitation

The spJCRISPR lines NT, 3A-S KD and 3A/3A-S KO were seeded in 8 14 cm dishes each, grown to confluency and differentiated. On Day 6 all plates were treated with 200 nM of the proteasome inhibitor Bortezomib (BTZ). On Day 7 (18 Hrs. post-BTZ) all plates were harvested in 4% SDS buffer containing protease and phosphatase inhibitors to ensure complete FOXO3A-Short extraction. Lysates were sheared with 21 then 23 gauge needles, centrifuged at 13,000 rpm for 5 minutes then quantitated with the DC Protein Assay kit (Biorad 5000116). Prior to the IP the lysates were diluted in RIPA buffer (1% Triton X-100, 1% sodium deoxycholate, 0.1% SDS, 150 mM NaCl, 20 mM Tris-HCl pH 7.4, 2 mM EDTA pH 8, 1 mM EGTA pH 8) to a final concentration of 0.5% SDS and precleared by nutating every 1 mg of protein with 50 µl of protein A agarose beads (Goldbio #P-400) overnight at 4°C. For each IP 25 µg of custom FOXO3A antibody, 1.5 mg of lysate and 50 µl of protein A agarose beads was used. Due to the molecular weight of FOXO3A-Short and low abundance nature of the protein we chose to do the IPs by crosslinking our antibody to the agarose beads with DMP to help eliminate heavy chain elution and subsequent detection. To do this beads were washed 3x in 0.1 M sodium borate pH 8.2 (SB) then incubated nutating at room temperature in SB with antibody for 1 hour. Beads were centrifuged

at 6000 rpm at RT for 1 minute, supernatant removed then washed 2x with SB then 2x with 0.2 M triethanolamine pH 8.2. A 60 mM solution of DMP (Thermo 21667) was made fresh in triethanolamine (crosslinking buffer) and incubated with the bead/antibody complex nutating at RT for 1 hour. The beads were spun down to remove the supernatant, washed 1x with 0.2 M ethanolamine pH 8.2 (quench buffer), spun down to remove supernatant then resuspended in ethanolamine and nutated at RT for 30 minutes. The beads were spun down to remove supernatant then washed 2x in 0.5 M NaCl to remove non-crosslinked antibody. The beads were then washed 3x with RIPA buffer. After the last wash beads were mixed with lysate and incubated overnight nutating at 4ºC. The next day the IPs were centrifuged at 6000 rpm for 3 minutes and the supernatant removed. The beads were washed 4x with RIPA buffer then resuspended in 30 µl 2x Laemmli buffer and boiled at 95-100ºC for 10 minutes. The beads were then spun down at 7000 rpm 1 minute at RT and the supernatant (IP eluate) collected. The eluate was then mixed with 7 µl of 6x loading buffer, boiled again then approximately 35 µl of eluate was loaded per well of a 10-well 8% gel. Western detection was done using 1:1000 dilution of Cell Signal FOXO3A #12829. To further eliminate trace heavy chain detection 1:1000 Cleanblot (Thermo 21230) was used as the secondary. Fempto ECL (Thermo 34095) was used for detection of FOXO3A-Short (usually 1 – 2 minute exposure). Pico ECL (Thermo 34580) was sufficient for FOXO3A detection.

Immunofluorescence

LHCNM2 myoblasts were seeded in 12-well plates and grown to confluency then differentiated and treated as necessary. After treatments they were fixed with 4% PFA for 15 minutes, permeabilized with 0.2% Triton X-100 for 5 minutes, washed 3x with PBS then incubated with a 1:1000 dilution of Cell Signal FOXO3A #12829 in 3% BSA TBST overnight at 4°C rocking. The next day primary antibody was removed and cells were stained with PBS containing 1:1000 Alexa Fluor 488 Rabbit secondary (Thermo A21206), 1:250 Alexa Fluor 568 Phalloidin (A12380) and 1:50,000 DAPI at RT for one hour. After washing with PBS cells were imaged on an EVOS FL Auto Cell Imaging System (Thermo).

Seahorse Assay

All Seahorse assays were performed on the XF96 Extracellular Flux Analyzer. LHCNM2 myoblasts were seeded at 16,000 cells/well in a 96-well Seahorse plate (collagen coated) in 200 µl of growth medium. One well in each corner of the plate was left containing medium only to serve as a background control during measurement. Three days after seeding differentiation was started by aspirating the growth medium, washing all wells 1x with 200 µl of PBS, aspirating and then applying 200 µl of differentiation medium per well. The medium was

changed every two days and differentiation was achieved on Day 6 or Day 7. On the day of the assay the medium was changed in all the wells to 150 µl of unbuffered Seahorse assay medium and the plate was maintained in non-CO_2 incubators from then on. The Seahorse assay medium was made by dissolving 8.3 grams of DMEM powder (Sigma D5030) in 900 ml MQ dH_2O, adding 16 mg phenol red (Sigma P5530), adding 1.85 g NaCl and adjusting the pH to 7.4 at 37 ˚C by the addition of +/- 190 µl 2 M NaOH and then bringing the volume up to 1000 ml. This medium was then filter sterilized. On the day of the assay this medium was supplemented with glucose to a final concentration of 25 mM (4.5 mg/ml) using 45% glucose solution (Sigma) and 2 mM glutamine. Equal numbers of wells per condition were equally distributed across the plate (alternating columns). For each assay both mitochondrial and glycolytic stress tests were performed. The protocol used consisted of 3 minute measurements with 2 minute mixes in between. It also contained injection points of 25 µl each per well per point. This created a typical profile of 6 measurements at baseline (including pre-incubated compounds) followed by the injection of 15 µM oligomycin, 6 measurements, injection of 10 µM FCCP, 5 measurements and then half the wells of each condition were either injected with a combination of 25 µM antimycin/12.5 µM rotenone or 1 M 2-deoxyglucose and this was followed by 3 or 5 final measurements. All of these compounds were purchased from Sigma and dissolved in DMSO, stored at -20 ˚C and then diluted in Seahorse medium except for 2-DG which was directly dissolved in Seahorse medium from powder on the day of the assay. Immediately after the 2 – 2.5 hour run was completed all the wells were incubated with 100 µl CyQuant Direct (Life Technologies) for 1 additional hour. The plate was then measured on a Biotek Synergy HT plate reader. For data analysis the XF^e software (version 2.1.0.162) was used. First the raw ECAR and OCR values were normalized to the CyQuant assay data, this was done to correct for any differences in cell number. This data was then exported from the program for further analysis. To analyze the ECAR we chose two representative time points within each assay; usually the 5th (baseline) and the 10th measurements (10th being 4 points after oligomycin injection). To establish the lower boundary of the ECAR measurement in each assay we averaged the values of the last measurement in the 2-DG injected wells from all conditions. This 2-DG average value was then subtracted from the individual baseline and post-oligomycin (ECAR max) values for each condition. Both the baseline and max ECAR 2-DG normalized values were then calculated as a percentage of the baseline of the absolute experimental control in each assay (i.e. the +Dox condition is normalized to the -Dox condition). Using the number of wells included in the analysis of each condition two-tailed Student's t-tests were then used to compare conditions and calculate p-values.

SUPPLEMENTAL REFERENCES

Banasik, K., Ribel-Madsen, R., Gjesing, A. P., Wegner, L., Andersson, A., Poulsen, P., … Vaag, A. (2011). The FOXO3A rs2802292 G-allele associates with improved peripheral and hepatic insulin sensitivity and increased skeletal muscle-FOXO3A mRNA expression in twins. *J. Clin. Endocrinol. Metab*, *96*(1), E119-E124. https://doi.org/10.1210/jc.2010-0881

Holmes, K., Williams, C. M., Chapman, E. A. & Cross, M. J. (2010). Detection of siRNA induced mRNA silencing by RT-qPCR: considerations for experimental design. *BMC Res. Notes*, *3*(1), 53. https://doi.org/10.1186/1756-0500-3-53

Meerbrey, K. L., Hu, G., Kessler, J. D., Roarty, K., Li, M. Z., Fang, J. E., … Elledge, S. J. (2011). The pINDUCER lentiviral toolkit for inducible RNA interference in vitro and in vivo. *Proc. Natl. Acad. Sci. U. S. A*, *108*(9), 3665-3670. https://doi.org/10.1073/pnas.1019736108

Santo, E. E., Ebus, M. E., Koster, J., Schulte, J. H., Lakeman, A., van Sluis, P., … Molenaar, J. J. (2012). Oncogenic activation of FOXR1 by 11q23 intrachromosomal deletion-fusions in neuroblastoma. *Oncogene*, *31*(12), 1571-1581. https://doi.org/10.1038/onc.2011.344

Santo, E. E., Stroeken, P., van Sluis, P., Koster, J., Versteeg, R. & Westerhout, E. M. (2013). FOXO3a is a major target of inactivation by PI3K/AKT signaling in aggressive neuroblastoma. *Cancer Res.*, *73*(7), 2189-2198. https://doi.org/10.1158/0008-5472.CAN-12-3767

Santo, E. E. & Paik, J. (2018). A splice junction-targeted CRISPR approach (spJCRISPR) reveals human FOXO3B to be a protein-coding gene. *Gene*, *673*), 95-101. https://doi.org/10.1016/j.gene.2018.06.048
